# Plant Drought Tolerance Enhancement by Trehalose Production of Desiccation-Tolerant Microorganisms

**DOI:** 10.3389/fmicb.2016.01577

**Published:** 2016-09-30

**Authors:** Juan I. Vílchez, Cristina García-Fontana, Desireé Román-Naranjo, Jesús González-López, Maximino Manzanera

**Affiliations:** Institute for Water Research, and Department of Microbiology, University of GranadaGranada, Spain

**Keywords:** desiccation tolerance, rhizobacterial drought-tolerance enhancers (RDTE), plant-growth-promoting rhizobacteria (PGPR), trehalose, *Pseudomonas putida* KT2440

## Abstract

A collection of desiccation-tolerant xeroprotectant-producing microorganisms was screened for their ability to protect plants against drought, and their role as plant growth-promoting rhizobacteria was investigated in two different crops (tomato and pepper). The most commonly described biochemical mechanisms for plant protection against drought by microorganisms including the production of phytohormones, antioxidants and xeroprotectants were analyzed. In particular, the degree of plant protection against drought provided by these microorganisms was characterized. After studying the findings and comparing them with results of the closest taxonomic relatives at the species and strain levels, we propose that trehalose produced by these microorganisms is correlated with their ability to protect plants against drought. This proposal is based on the increased protection of plants against drought by the desiccation-sensitive microorganism *Pseudomonas putida* KT2440, which expresses the *ots*AB genes for trehalose biosynthesis *in trans*.

## Introduction

Drought, the main limiting factor for crop yields worldwide (Sharp et al., 2004), is considered the abiotic stress with the greatest effect on plants, since 45% of global farming lands are subjected to continuous or frequent droughts (Long and Ort, 2010). The Intergovernmental Panel on Climate Change (IPCC) has projected that the land area affected by drought will increase while water resources in the affected areas could decline by as much as 30% by mid-century (Christensen et al., 2007).

In this context, ethylene, abscisic acid (ABA) and indoleacetic acid (IAA) are phytohormones often associated with plant senescence or root control (Stella et al., 1996) and have also been associated with how plants respond to water deficit (Yamaguchi-Shinozaki and Shinozaki, 1994; Kawakami et al., 2010). Ethylene is synthesized from 1-amino cyclopropane-1-carboxylic acid (ACC) by the enzyme ACC oxidase (ACO or ethylene-forming enzyme; EC:1.14.17.4), and regulates a number of physiological mechanisms. At low concentrations, it promotes the development of adventitious roots and root hairs (Nadeem et al., 2010), and at high concentrations it inhibits root growth under stress conditions such as drying soil (Glick et al., 2007). With regard to ABA, this key phytohormone causes stomatal closure to prevent further decreases in water content, promotes root growth and the accumulation of compatible solutes, and regulates the synthesis of dehydrins as well as late embryogenesis abundant (LEA) proteins, thus coordinating various facets of the plant’s response to low water potential (ψ_w_) (Pierce and Raschke, 1980; Zhu, 2002; Raschke, 2006). Some auxins, including IAA, when present in low concentrations, increase cell elongation, resulting in greater root growth and the generation of lateral roots. However, at higher concentrations IAA, inhibits root growth either directly or indirectly via the promotion of ethylene synthesis. The effect of ethylene is intertwined with the production of IAA, since on the one hand ethylene has been shown to lower endogenous IAA levels in different plant tissues, and on the other hand IAA stimulates ethylene production by promoting ACC synthase activity (Glick, 2003; Dimkpa et al., 2009).

The accumulation of xeroprotectants such as trehalose by some microorganisms and some plants enables them to withstand extreme abiotic stress such as desiccation (Chaplin, 2006; Julca et al., 2012). Efforts have been made to bolster plant drought tolerance by developing transgenic plants with altered phytohormone production (Sobeih et al., 2004) and greater trehalose concentrations (Goddijn and Smeekens, 1998; Pilon-Smits et al., 1998). In addition to transgenic plants, plant-growth-promoting rhizobacteria (PGPR) have great potential in agriculture since their presence in the roots increases crop production by enhancing the plant’s tolerance to drought and other types of environmental stress (Vilchez and Manzanera, 2011). According to Timmusk et al. (2013), these bacteria are called rhizobacterial drought-tolerance enhancers (RDTE). Over the last decade, RDTE have received particular attention (Glick et al., 2007), and since Mayak et al. (2004) reported that the strain *Achromobacter piechaudii* ARV8 was useful in protecting plants against drought, many other microorganisms have been described with similar properties. In general, these microorganisms are able to counteract the negative effects of drought stress in plants by producing ACC deaminase (ACCd), lowering ABA, or raising IAA production (Mayak et al., 2004). See Kaushal and Wani (2016) for a recent review on this subject.

Water stress also affects the viability of microorganisms in different ways (Manzanera et al., 2002) by compromising protein structure, stability, activity, folding, and assembly by disrupting the physical architecture and functions of nucleic acids; and by altering membrane structure and function (Chaplin, 2006; Raschke, 2006; Julca et al., 2012). A large number of RDTE have been isolated in samples from dry or drought-affected locations (Mayak et al., 2004; Dimkpa et al., 2009; Roca et al., 2013), thus suggesting that this type of microorganism might be well adapted to water stress.

Drought conditions would therefore be expected to produce the simultaneous selection of tolerant plants and microorganisms in the environment, assuming that the selection of desiccation-tolerant microorganisms counteracts the deleterious effect of drought on plants (Lau and Lennon, 2012). However, no study available has drawn a clear connection between a microbial phenotype and the ability to protect plants from drying. The present study establishes a correlation between tolerance to desiccation in a set of highly desiccation-tolerant PGPR and their closest taxonomic relatives at the species and strain level, and the level of plant protection against drought in two different crops (tomato and pepper plants). This correlation between desiccation tolerance of the microorganism and its ability to protect some plants from drought seems to depend on the microorganism’s capacity to finely regulate the concentration of trehalose in the plant as a signal of drying damage, since trehalose triggers the plant-defense system to counteract the damage caused by drought. To demonstrate the role of microbial trehalose in protecting the plant against desiccation, we isolated the *ots*AB genes, coding for alpha, alpha-trehalose-phosphate synthase and trehalose-6-phosphate phosphatase, from the highly desiccation-tolerant strain *Microbacterium sp.* 3J1, and inserted them into the desiccation-sensitive microorganism *P. putida* KT2440, generating *P. putida* KT2440 (pUCP22:*ots*AB) with a significant increase in the intracellular concentration of trehalose under water stress conditions. In addition, plants inoculated with the resulting trehalose-overexpressing *P. putida* KT2440 (pUCP22:*ots*AB) showed a significantly greater tolerance to drought.

## Materials and Methods

### Microorganisms, Media, and Culture Conditions

The strains used in this study are shown in **Table 1**. The organisms described here will be made available upon request. Bacteria were grown in tryptic soy broth (TSB) or M9 Minimal Medium (Sigma M6030) with glucose or fructose (50 mM) as the sole carbon source at 30°C (Manzanera et al., 2004). To prepare hypersaline minimal medium (HMM), we added NaCl (6 M) to M9 medium at the concentration specified by Manzanera et al. (2002). To generate hyperosmotic conditions, we added 5% or 50% (wt/vol) polyethylene glycol (PEG) 6000 was added to the media (Sandhya et al., 2009).

**Table 1:** Viability of different bacterial isolates after 24 h of air drying.

Strain	DSMZ No.	Reference	Survival rate mean (%) ±SD^a^
*P. putida* KT2440		Nelson et al., 2002	0 ± 0
*Microbacterium sp.* 3J1		Narváez-Reinaldo et al., 2010	42.52 ± 7.21
*Rhodococcus sp.* 4J2A2		Narváez-Reinaldo et al., 2010	25.99 ± 5.52
*Leucobacter sp.* 4J7B1		Narváez-Reinaldo et al., 2010	17.81 ± 3.53
*Arthrobacter siccitolerans* 4J27		Narváez-Reinaldo et al., 2010	18.1 ± 2.60
*A. koreensis* 5J12		Narváez-Reinaldo et al., 2010	29.4 ± 4.68
*A. luteolus* CF25	13067	Wauters et al., 2000	1.91 ± 0.02
*A. koreensis* CA15-8	16760	Lee et al., 2003	12.57 ± 1.68
*M. foliorum* P333/02	12966	Behrendt et al., 2001	25.06 ± 4.89
*M. phyllosphaerae* P369/06	13468	Behrendt et al., 2001	8.9 ± 0.04
*Achromobacter piechaudii*	366-5	Kiredjian et al., 1986	24.6 ± 5.34


*SD^a^ – The data are the means and standard deviations of three independent determinations.*

### Air Drying: Determination of Survival Rates

A colony of each pure culture containing 10^7^–10^9^ cells was suspended in 1 mL M9 Minimal Medium. Aliquot fractions (100 μL) were placed on sterile Petri dishes and dried under a current of sterile air for 24 h. Cells were suspended in 1 mL sterile saline buffer, and serial dilutions of the cells before and after drying were plated on trypticase soy agar (TSA) plates. All the experiments were performed at room temperature. The survival rate was calculated as CFU/mL after drying with reference to the pre-drying CFU/mL, expressed as a percentage. The assays were performed in triplicate following to Narváez-Reinaldo et al. (2010).

### Plant Material and Growth Conditions

Tomato (*Lycopersicum esculentum* Mill *cv*. F144) and green pepper (*Capsicum annuum* L. *cv.* Maor) seedlings were started from sterile seeds that were sown in plastic trays in wet vermiculite accordingly to Mayak et al. (2004). After 1 week, uniform-sized seedlings (shoot height approximately 3 cm) were selected and planted in non-sterile soil composed of a mixture of plant substrate (black peat, vegetable compost, white peat, and coconut pH 7.2 and 56% organic matter) and vermiculite (1:1), one per pot, using 0.4 L pots filled with approx. 0.26 L of soil mixture. The pots were incubated in a Climates Ing GROW growth chamber at constant relative humidity (50–60%). The chamber was lit with a 12-h day/night cycle and gradual dimming/brightening of the light to simulate dawn and dusk. The day cycle consisted of 200 μmol photons⋅m^-2^⋅s^-1^, and the dawn–dusk cycle consisted of 150 μmol photons⋅m^-2^⋅s^-1^. The temperature was programmed to change from 18 to 20°C for the night cycle to 20–25°C in the diurnal cycle. Seedlings were regularly watered during the first 2 weeks. On day 7, after being transferred to the pots, the plants were fertilized with basal salt Murashige and Skoog medium diluted 1/10 (vol/vol) (Murashige and Skoog, 1962) and on day 14, they were inoculated with the bacterial strains. This time was considered experimental day 0 for plant growth-promotion tests.

At inoculation, seedlings were treated with 40 mL of bacterial suspension (10^8^–10^9^ CFU/mL) in sterile M9 buffer, and non-inoculated controls were watered with sterile M9 buffer. Inoculated and non-inoculated plants were watered for 2 weeks after the seedlings were transplanted.

### Monitoring of Plant Growth

After 2 weeks, watering with the buffer was stopped, and this time was considered time 0 for the drought-tolerance-enhancement tests. Thus, dry weight (DW), fresh weight (FW), fully turgid weight (FTW) of the whole plants free from soil were measured four times after inoculation at 7, 14, 21, and 33 days. The relative water content (RWC) was calculated according to Mayak et al. (2004) as follows: RWC = (FW-DW) × (TW-DW)^-1^. In addition, root length (RL) and stem length (SL) were recorded.

### Trehalose Production by Bacterial Strains

Intracellular trehalose was determined following to Manzanera et al. (2002) with slight modifications. Samples of 1 mL were aliquoted from cultures in the exponential growth phase, and were then centrifuged and washed in 1 × M9 minimal medium without a carbon source. The bacterial pellets were lysed with FastPrep, and the trehalose concentration was determined by ion chromatography in a high-performance 940 Professional IC Vario 2 chromatography system (Metrohm, Switzerland) with a MetroSTEP Carb 2 copolymer polystyrene-divinylbenzene column (with quaternary ammonium groups) measuring 250 mm by 4.0 mm (Metrohm, Switzerland). The column temperature was set at 30°C at a pressure of 130 atmospheres (1911 psi). Trehalose concentrations were calculated relative to a reference standard curve, and efficiency of the process relative to sucrose detected was calculated with reference to the viable cells counted in the initial cultures.

### 16S rRNA Gene Analysis

To identify the most closely related bacterial species to the desiccation-tolerant strain collection, the nearly complete sequence of 16S rRNA from the different isolates was aligned with the sequences in closely related species. A phylogenetic tree of alignments was generated with the CLUSTAL X 2 program (Larkin et al., 2007), and phylogenetic trees were inferred with the neighbor-joining and maximum-likelihood methods using the MEGA 5.0 software package (Tamura et al., 2011). Bootstrap analysis was based on 1,000 resamplings. The distances were calculated according to Kimura’s two-parameter model.

### Construction of the Plasmid-Expressing *ots*AB

To construct an *ots*AB-expressing plasmid, a 2.291-kb *ots*AB gene fragment from *Microbacterium* sp. 3J1 was amplified with the oligonucleotides OtsAB-F (5′AGGAATTCCACCCATGCCAGCCGCA 3′) and OtsAB-R (5′TCAAGCTTGGACATGACGAGAGAGTCTATTCCCG 3′). Oligonucleotides were designed with the help of Clone Manager software, and the 30-cycles PCR program included 30 s denaturation steps at 94°C, followed by 30 s annealing steps at 55°C and 150 s extension steps at 72°C. The *Eco*RI and *Hind*III sites, which cut into the polylinker of the vector, but not into the insert sequence, were included in the oligonucleotides (underlined). The plasmid was then cloned into pUCP22 to obtain the vector pUCP22:*ots*AB in *Escherichia coli* DH5α (West et al., 1994). The sequence of the resulting construct was analyzed by Sanger sequencing, and *P. putida* KT2440 was transformed with both plasmids (pUCP22 and pUCP22:otsAB) by electroporation, as described previously (Enderle and Farwell, 1998). Trehalose production by the transformed strains was analyzed as described in the preceding section (Trehalose production by bacterial strains).

### Superoxide Dismutase and Catalase Activity of Bacterial Strains

Superoxide dismutase activity was determined according to Beauchamp and Fridovich (1971).

In addition, CAT activity of the strains was determined with the method of Aebi (1984) by measuring the decomposition of H_2_O_2_ directly as the decrease in absorbance at 240 nm.

### Indoleacetic Acid, Abscisic Acid, and Gibberellic Acid Production by Strains

The production of IAA by bacterial strains was determined following to Bano and Musarrat (2003). Abscisic acid and gibberellic acid (GA_3_) production were detected and measured with thin-layer chromatography (TLC) and quantified by high-performance liquid chromatography (HPLC) as in Karadeniz et al. (2006), with slight modifications taken from Ross et al. (1987). Strains were grown in TSB at 120 rpm and 30°C until they reached the exponential growth phase. Next, 100 mL of the cultures were extracted and centrifuged at 4,800 × *g* for 15 min, and the samples were filtered to sterilize the supernatants. They were supplemented with 1 mL butylated hydroxytoluene (BHT) to prevent hormone oxidation. Extraction was done with ethyl acetate and concentration in a rotary evaporator prior to TLC. Chromatography was performed in a mixture of isopropanol:ammonia:distilled water (10:1:1, vol/vol/vol) on silica gel TLC plates (60F254 TLC, Merck).

### Salicylic Acid Production and Aminocyclopropane Carboxylic Acid Consumption by Strains

Qualitative determination of salicylic acid (SA) by TLC followed to the protocol of Buysens et al. (1996), and quantitative determinations were performed by colorimetry. The consumption of ACC by bacterial strains was determined as in Penrose and Glick (2003).

### Data Analyses

Analysis of covariance (ANCOVA) was used to determine whether desiccation tolerance of plants determined as FW, DW, FTW, RWC and the size of plants subjected to drought considered as plant responses (continuous predictive variables or covariates) was influenced by bacterial activity. This activity was measured as the concentration of phytohormones, antioxidants, and trehalose (dependent variables) produced by the different microorganisms. Prior to the ANCOVA test, Levene’s test for equality of variances was performed. When Levene’s test was positive (*P* < 0.05) the variances in the groups were considered different, and therefore the assumptions for the ANCOVA were not met. Pearson correlation coefficients were calculated to investigate the relationship between plant variables and microbial activity.

## Results and Discussion

**FIGURE 1:**
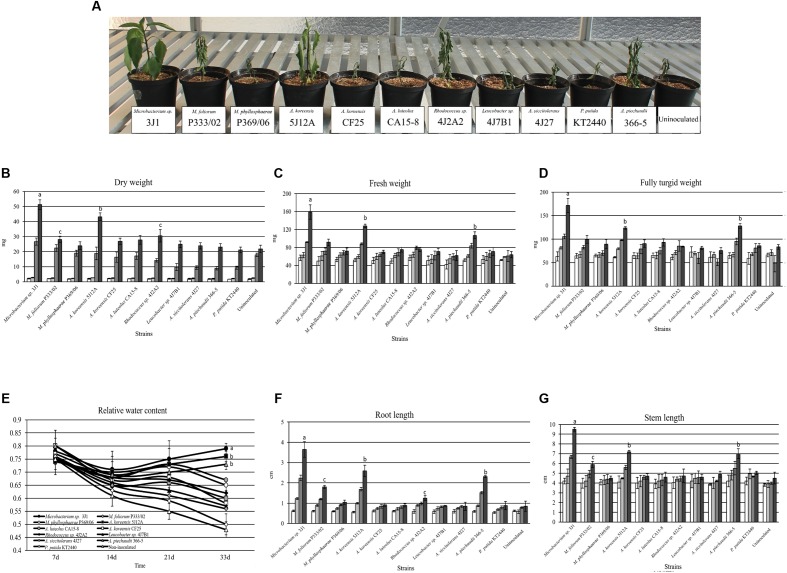
**The effect of desiccation-tolerant microorganisms on pepper plants subjected to water stress.** **(A)** Shows the physical appearance of pepper plants after 33 days without watering. **(B,C)** show the DW and FW (mg), respectively, of whole pepper plants free from soil. **(D–G)** show the FTW (mg), RWC, RL, and SL, respectively, of whole pepper plants free from soil. White bars correspond to day 7, light-gray bars correspond to day 14, dark bars correspond to day 21 and black bars correspond to day 33. Values are the means of three replicates ±SD.

Pepper and tomato plants were used to test the ability of five highly desiccation-tolerant microorganisms to protect plants against drought. All five bacterial strains were previously isolated in our laboratory: *Microbacterium* sp. 3J1, *Arthrobacter koreensis* 5J12A, *A. siccitolerans* 4J27, *Rhodococcus* sp. 4J2A2, and *Leucobacter* sp. 4J7B1 (Narváez-Reinaldo et al., 2010). For a negative control, we used the desiccation-sensitive PGPR *P. putida* KT2440, and for a positive control, we used the well-characterized RDTE *A. piechaudii* 366-5 (Manzanera et al., 2002; Dos Santos et al., 2004; Mayak et al., 2004). An additional set of non-inoculated plants was used as a negative control. Plants were inoculated and subjected to drying conditions as described in the experimental procedures section. After 33 days in the absence of water, both non-inoculated pepper plants and plants inoculated with desiccation-sensitive *P. putida* KT2440 showed signs of dying (**Figure 1A**). Similar results were seen when the plants were inoculated with *A. siccitolerans* 4J27, *Leucobacter* sp. 4J7B1 and *Rhodococcus* sp. 4J2A2. However, pepper plants inoculated with the most desiccation-tolerant isolates, *Microbacterium* sp. 3J1 and *A. koreensis* 5J12A, as well as with *A. piechaudii* 366-5, appeared healthy and yielded the highest values of DW (**Figure 1B**), FW (**Figure 1C**), FTW (**Figure 1D**), and RWC (**Figure 1E**), and had the longest roots (**Figure 1F**) and stems (**Figure 1G**) at the end of the experiment. Soil moisture was reduced from 71.52 to 13.75%. No statistical differences were found in the soil moisture regardless of the applied strain applied. Analogous results were found when tomato plants were used, thus validating these findings in a different plant species (**Supplementary Figure S1**). However, some minor but significant protection against drought was detected in tomato plants when *Rhodococcus* sp. 4J2A2 was used as the inoculant. These results suggest that some plant species are more sensitive than others to certain xerotolerant strains, since *Rhodococcus* sp. 4J2A2 showed a xeroprotectant effect in tomato but not in pepper plants under our assay conditions. In addition, these results suggest that only the strains showing the highest desiccation tolerance protect certain plants against drought.

**FIGURE 2:**
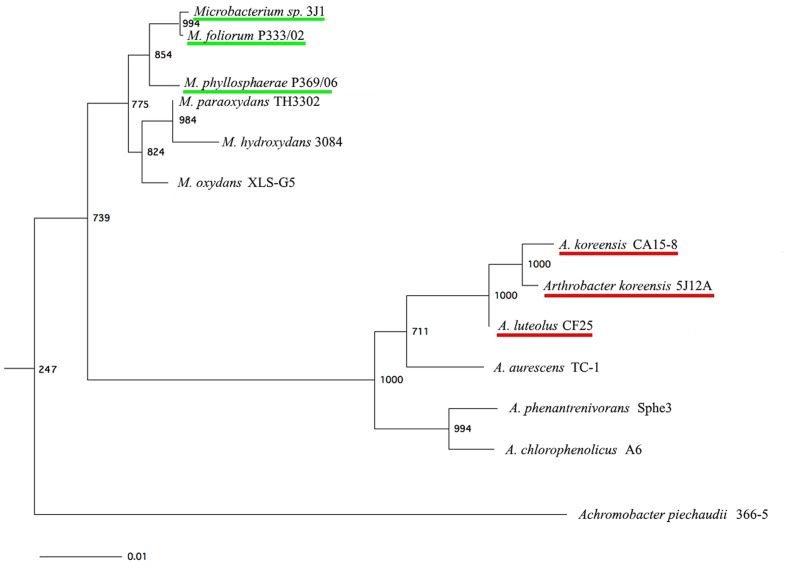
**Neighbor-joining phylogenetic tree based on the 16S rRNA sequence of strains *Microbacterium* sp. 3J1 and *A. koreensis* 5J12A (both with the latter’s closest relative strains), and *A. piechaudii* 366-5, used as outgroup.** Numbers at the bifurcations indicate how many times each species occurred at this position. Bar, 0.01 changes per nucleotide position.

To determine whether this protective effect was a specific characteristic of the isolate itself or was a property shared by strains that are closely related taxonomically, we compared the sequence for the 16S rRNA gene in strains *Microbacterium* sp. 3J1 (GenBank accession number GU815136) and *A. koreensis* 5J12A (GenBank accession number GU815140; Narváez-Reinaldo et al., 2010) with the sequence from the EzTaxon server corresponding to the same gene^1^ in order to identify the most closely related strains. The nearly complete sequence for the 16S rRNA gene in strains *Microbacterium* sp. 3J1 and *A. koreensis* 5J12A (approximately 1,500 bp) was aligned with the sequences in closely related species of the genera *Microbacterium* and *Arthrobacter*, including *A. piechaudii* ATCC 43552 as an outgroup. The resulting neighbor-joining trees are shown in **Figure 2**.

Once, we determined their taxonomic affiliations, the bacterial strains identified as most closely related to *Microbacterium* sp. 3J1 were *M. foliorum* P333/02 (Behrendt et al., 2001) and *M. phyllosphaerae* P369/06 (Behrendt et al., 2001), while the strains found to be most closely related to *A. koreensis* 5J12A proved to be *A. koreensis* CA15-8 (Lee et al., 2003) and *A. luteolus* CF25 (Wauters et al., 2000). Further comparisons showed that the desiccation tolerance provided by *Microbacterium* sp. 3J1 and *A. koreensis* 5J12A appeared to correlate with the degree of drought protection they provided to the plant. In other words, plants were most tolerant to drought when they were inoculated with *Microbacterium* sp. 3J1 and *A. koreensis* 5J12A, according to the high values, we found for desiccation-tolerance parameters. The intermediate levels of desiccation tolerance provided by *M. foliorum* P333/02, *M. phyllospherae* P369/06, *A. koreensis* CA15-8, and *A. luteolus* CF25 were translated into intermediate levels of protection of the plant against drought.

**FIGURE 3:**
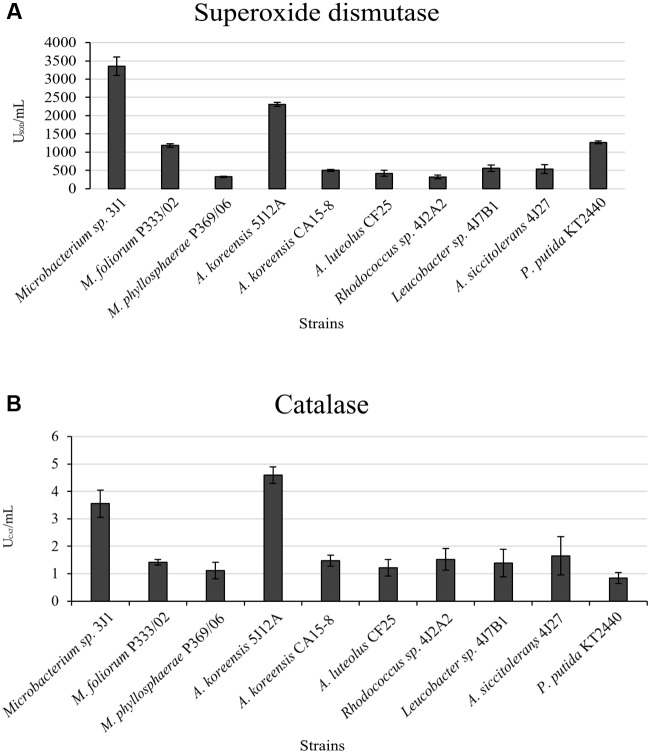
**Antioxidant enzyme activity in different strains.** **(A)** shows SOD in U_SOD_/mL; and **(B)** shows CAT in U_CAT_/mL (units of enzyme activity, U). *Pseudomonas putida* KT2440 was used as the control strain. Values are the means of three replicates ±SD.

**FIGURE 4:**
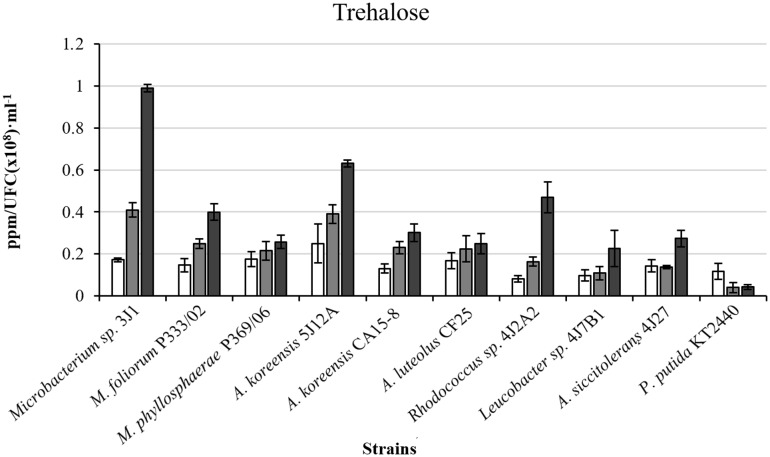
**Intracellular trehalose production in ppm/UFC (×10^8^)⋅mL^-1^ by bacterial strains in different polyethylene glycol (PEG) concentrations.** White bars correspond to cultures in the absence of PEG (0% PEG); gray bars, correspond to cultures supplemented with 5% PEG; and black bars, to cultures supplemented with 50% PEG. *P. putida* KT2440 was used as the control strain. Values are the means of three replicates ±SD.

**FIGURE 5:**
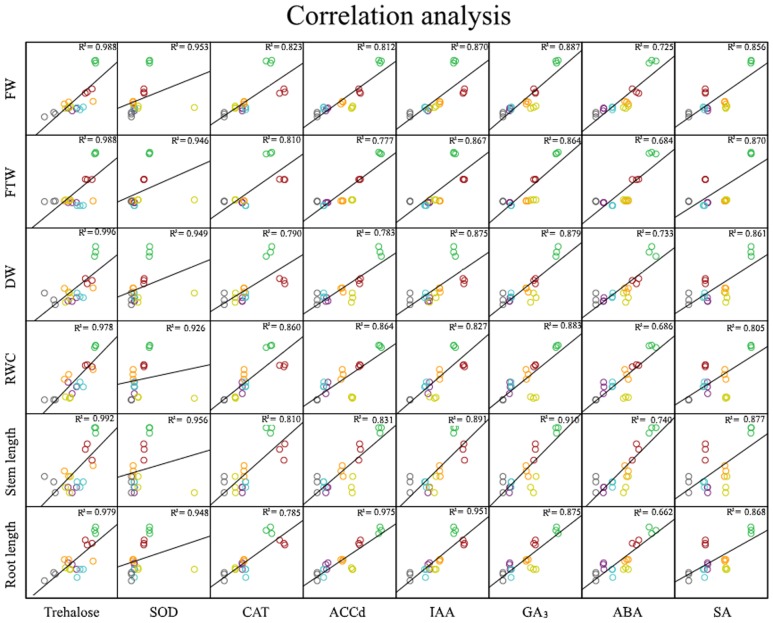
**Analysis of statistical correlation.** One-way ANCOVA for the correlations between production values by each strain of trehalose, SOD, CAT, ACCd, IAA, GA_3_, ABA, SA, and values for plant DW, FW, FTW, RWC, shoot length, and root length. Green circles correspond to *Microbacterium* sp. 3J1; red circles correspond to *A. koreensis* 5J12A; orange circles correspond to *Rhodococcus* sp. 4J2A2; violet circles correspond to *Leucobacter* sp. 4J7B1; blue circles correspond to *A. siccitolerans* 4J27; yellow circles correspond to *P. putida* KT2440, and gray circles correspond to non-inoculated samples.

In light of our results and earlier findings in the genera studied here, we suggest a possible correlation between the degree of desiccation tolerance in a given PGPR and the level of protection against drought it confers to the plant. To identify the molecule responsible for plant protection, we tested the strategies most commonly used by RDTE. Our panel of assays tested the *in vitro* production of phytohormones by the microorganisms involved in protection against desiccation (IAA, GA_3_, ABA, and SA) as well as ACC consumption (**Table 2**), antioxidant molecules involved preventing and repairing damage caused by reactive oxygen species (ROS) [superoxide dismutase (SOD) and catalase (CAT)] (**Figure 3**), and trehalose as a xeroprotectant involved in the conservation of essential biomolecules (**Figure 4**). Our ANCOVA (one-way ANOVA, *p* < 0.05) results showed a correlation between plant-growth parameters, e.g., DW, FW and FTW, RWC, RL, and shoot length (SL), and the concentration of trehalose produced by the microbial cells (**Figure 5**). This analysis indicates that the highest trehalose production by the strain *Microbacterium sp.* 3J1 correlated strongly with the highest values of DW, FW, RWC, and RL in plants inoculated with this strain, as documented by coefficients of determination, R^2^ above 0.8. Moreover, trehalose production by *P. putida* KT2440 (a desiccation-sensitive strain) and the rest of the analyzed strains analyzed showed similar correlations, which indicate that the values of all these parameters depend on trehalose production. This molecule which can stabilize essential biomolecules in plant cells, has been linked to the modulation of plant metabolism via the induction of stress-response genes, mostly transcriptional factors and protein kinases (Schluepmann et al., 2004). Bae et al. (2005) found that 30 mM trehalose, when added exogenously, was linked to the regulation of ethylene- and jasmonate-signaling pathways. The presence of trehalose thus appears to lead to cross-talk with ABA signaling in different physiological processes, including the regulation of stomatal aperture size (Gómez et al., 2010). Inoculation of *Medicago truncatula* with an IAA-overproducing strain of *Sinorhizobium meliloti* led to the accumulation of trehalose in the bacterial cell, and re-modulation of the plant’s phytohormone profile (Bianco and Defez, 2009). These results are evidence of a two-way interaction system between trehalose as the elicitor produced by the bacteria and the phytohormone regulatory map of the plant. In addition, exogenously added trehalose has been associated with protection against oxidative damage by reducing ROS accumulation, increasing non-enzymatic antioxidants, and co-activating the antioxidative and glyoxalase system (Mostofa et al., 2014). A more in-depth discussion of these bi-directional regulatory mechanisms was published by Lunn et al. (2014) in a recent review of trehalose metabolism in plants (Lunn et al., 2014).

**Table 2:** Production of phytohormones by different strains.

Strains	AIA	ABA	GA	SA	ACCd
*Microbacterium* sp. 3J1	26.8 ± 2.5	1.64 ± 0.12	16.3 ± 1.3	95.6 ± 7.4	0.1447 ± 0.0026
*A. koreensis* 5J12A	33.5 ± 1.1	1.12 ± 0.02	9.5 ± 0.7	25.4 ± 5.6	0.0964 ± 0.0035
*Rhodococcus* sp. 4J2A2	18.1 ± 2.8	0.89 ± 0.02	7.1 ± 0.5	55.6 ± 3.5	0.0584 ± 0.0015
*Leucobacter* sp. 4J7B1	12.0 ± 1.6	0.25 ± 0.04	2.2 ± 0.1	28.9 ± 5.8	0.0216 ± 0.0031
*A. siccitolerans* 4J27	9.5 ± 1.5	0.52 ± 0.10	2.5 ± 0.5	24.4 ± 7.2	0.0153 ± 0.0028
*P. putida* KT2440	18.0 ± 2.6	1 ± 0.21	10.2 ± 1.0	58.9 ± 1.4	0.0824 ± 0.0051


*Production of IAA, ABA, GA_*3*_, SA, and activity of ACCd (E) are shown in μg/mL. *P. putida* KT2440 was used as the control strain. Values are the means of three replicates ±SD.*

**FIGURE 6:**
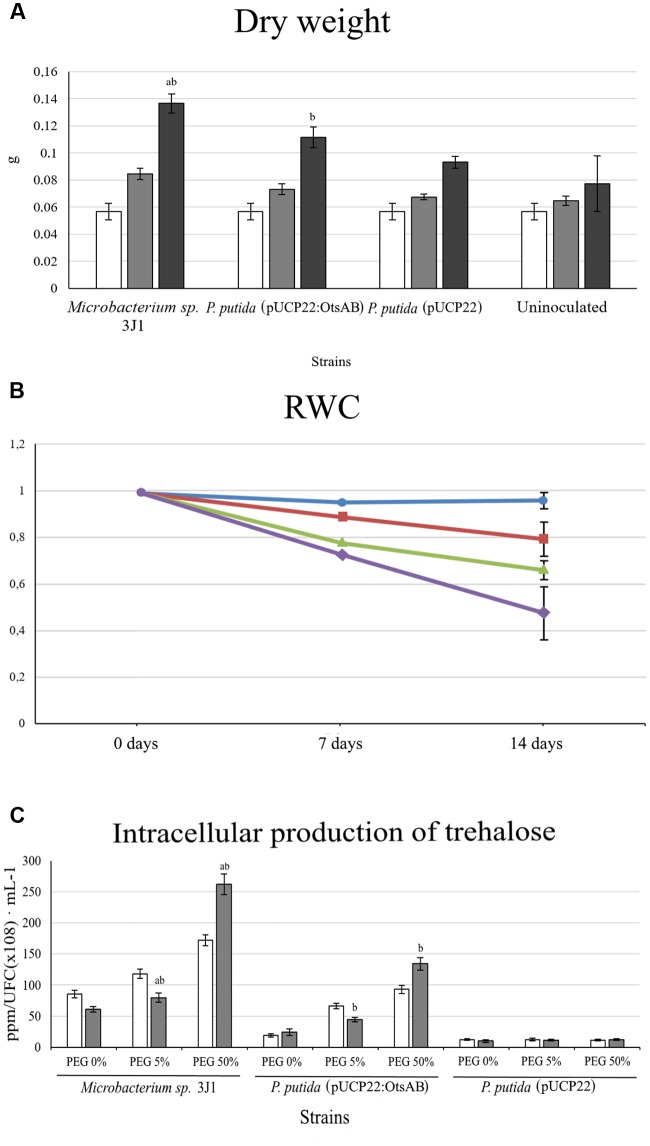
**Trehalose production by *P. putida* KT2440 containing the *ots*AB genes from *Microbacterium* sp. 3J1 and effect over pepper plants.** **(A)** Shows the DW of pepper plants inoculated with *P. putida* KT2440 (pUCP22), *P. putida* (pUCP22:otsAB) and *Microbacterium* sp. 3J1. Where white bars correspond to day 0, light-gray bars correspond to day 7, black bars correspond to day 14. Values are the means of three replicates ±SD. **(B)** Shows the relative water content of pepper plants inoculated with *P. putida* KT2440 (pUCP22; triangles), *P. putida* (pUCP22:otsAB; squares), and *Microbacterium* sp. 3J1 (circles), as well as non-inoculated plants (diamonds) at time 0, 7, and 14 days. Values are the means of three replicates ±SD. **(C)** Shows the intracellular concentration of trehalose in the absence of PEG and in presence of 5 and 50% PEG. White bars correspond to cultures grown on M9 minimal medium, and gray bars to cultures grown on TSB medium. All bars show SD; ^ab^indicates a significant difference with respect to all other results; ^b^indicates a significant difference with respect to control.

Salt stress in plants is known to induce the accumulation of ROS species such as H_2_O_2_. To avoid or alleviate the risk of ROS accumulation, plants respond by activating different scavenging pathways (Fujita et al., 2006). Among the scavenging systems, SOD is the primary scavenger in the detoxification of ROS induced by oxidative stress in plants. Other scavenging enzymes, including the ascorbate–glutathione cycle enzymes (ascorbate peroxidase and glutathione reductase) and CAT, also play an important role in detoxifying of the toxic cellular products of SOD (i.e., H_2_O_2_) in plant cells (Mittler, 2002). Moreover, exogenous trehalose is able to differentially modulate antioxidant enzymes and the expression of related plant genes (Nounjan et al., 2012). To elucidate the role of trehalose production by the microorganisms in protecting the plant against desiccation applied to the plant, we assayed the effect of *ots*AB genes from *Microbacterium* sp. 3J1 that were expressed in *P. putida* KT2440 under the constitutive expression of the P*lac* promoter in the broad-host-range vector pUCP22 in order to generate the pUCP22:*ots*AB vector (West et al., 1994). The trehalose concentration in *P. putida* KT2440 (pUCP22:*ots*AB) increased more than one order of magnitude (from 12.12 to 134.14 ppm/CFU(10^8^)⋅ml^-1^) compared to the concentration in *P. putida* KT2440 (pUCP22), and was nearly half the trehalose production by *Microbacterium* sp. 3J1 when grown in the presence of PEG 50% (-2.64 MPa). In addition, when we compared the desiccation tolerance of pepper plants inoculated with the trehalose-overproducing *P. putida* KT2440 (pUCP22:*ots*AB) to plants inoculated with *P. putida* KT2440 (pUCP22), we found a significant increase in desiccation tolerance in the former based on the ANOVA test (*p* < 0.05). DW of the plants inoculated with *P. putida* KT2440 (pUCP22:*ots*AB) was 1.13- and 1.2-fold higher than for plants inoculated with *P. putida* KT2440 (pUCP22) and uninoculated plants, respectively, while we observed an increase in RWC of 1.21-fold compared to plants inoculated with *P. putida* KT2440 (pUCP22) and 1.68-fold compared to non-inoculated plants (**Figure 6**).

These results point to a possible link between the degree of desiccation tolerance imparted by trehalose production by a given PGPR and the level of protection against drought that the organism confers to plants. Isolation assays have been performed to select RDTE from different locations such as quarry sand and riverside sand. All genera isolated in these studies belonged to *Actinobacteria*, one of the groups with the highest desiccation-tolerance values. This characteristic may reflect their natural abundance in soils, which may be explained by the broader tolerance of this genus to abiotic stress such as drought, extreme cold and starvation (Deutch and Perera, 1992; Zevenhuizen, 1992). We speculate that the high desiccation tolerance of these microorganisms enables the survival of a sufficient number of cells to colonize and establish interactions with the plant prior to the establishment of the symbiotic protection of the plant.

This is the first report available to link desiccation tolerance of a microorganism to the protection they confer to plants against drought. Plant-survival rate seems to correlate with the ability of the microbial cell to produce trehalose. The trehalose produced by the microorganism may control most of the plant’s enzymatic and non-enzymatic responses by favoring the production of the plant’s repertoire of phytohormones.

## Conclusion

We propose that the ability of microorganisms to produce trehalose under drying conditions can facilitate their own survival and that of the plant being colonized. This makes the selection of highly desiccation-tolerant PGPR potentially useful in agriculture as biostimulants to protect plants against drought.

## Author Contributions

JV, CG-F, and DR-N performed the experimental assays. JV, JG-L, and MM have performed the statistical analysis. JV and MM have designed and written the article.

## Conflict of Interest Statement

The authors declare that the research was conducted in the absence of any commercial or financial relationships that could be construed as a potential conflict of interest.

## Footnotes

1 http://eztaxon-e.ezbiocloud.net/

## Supplementary Material

**FIGURE S1 | The effect of desiccation-tolerant microorganisms on tomato plants subjected to water stress.** **(A)** Shows the physical appearance of pepper plants after 33 days without watering. **(B–D)** show the DW, FW and FTW (mg), respectively, of whole tomato plants free from soil. **(E–G)** show the RWC, RL and SL, respectively, of whole tomato plants free from soil. White bars correspond to day 7, light-gray bars correspond to day 14, dark bars correspond to day 21 and black bars correspond to day 33. Values are the means of 3 replicates ± SD.
