# Supplementary figures and images for: Plant Drought Tolerance Enhancement by Trehalose Production of Desiccation-Tolerant Microorganisms
Source: Front Microbiol. 2016 Sep 30;7:1577. doi: 10.3389/fmicb.2016.01577 (PMC5043138; doi:10.3389/fmicb.2016.01577)

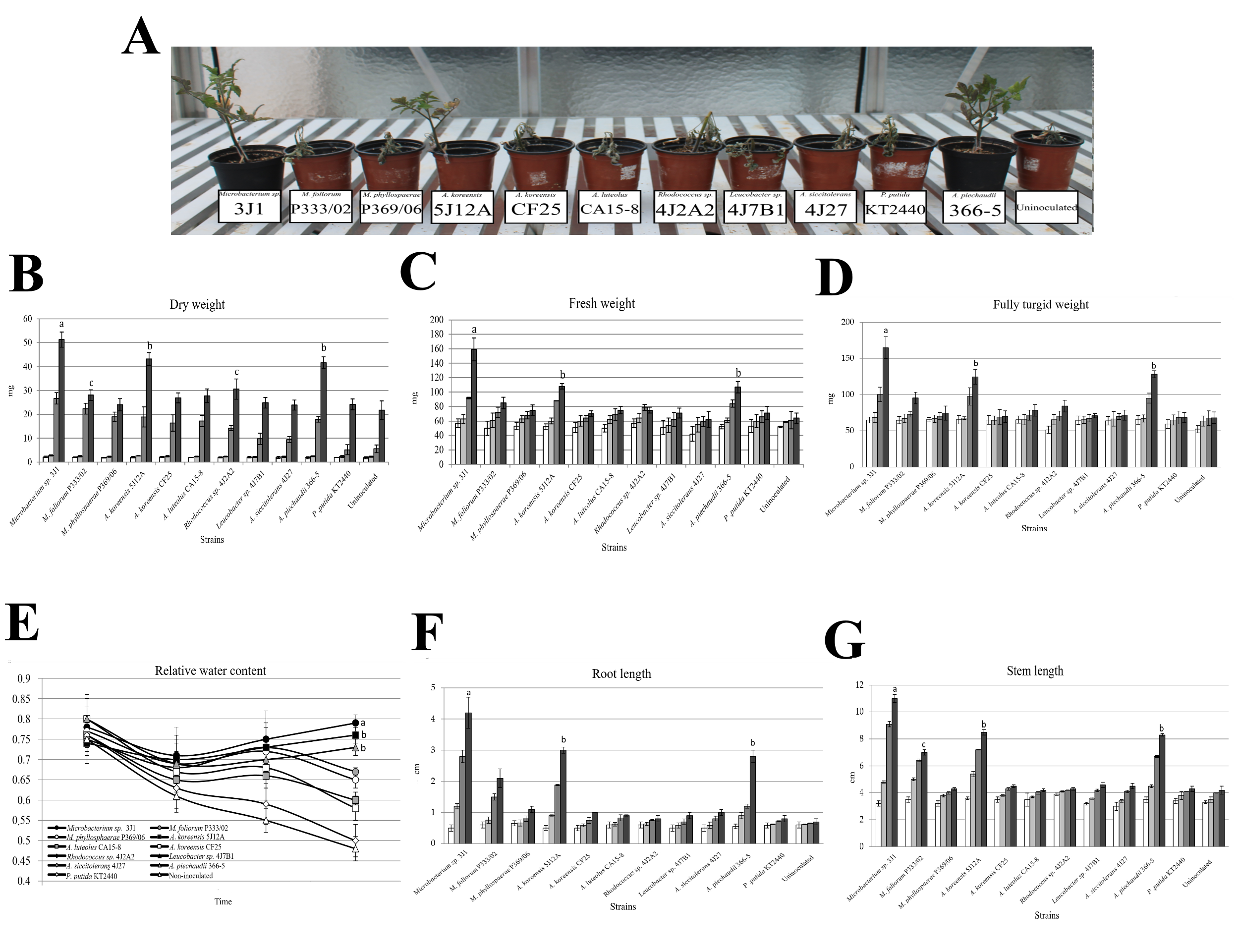

Supplement: Supplementary file 1 [file Image_1.TIF]
